# Editorial Notes

**Published:** 1894-04

**Authors:** 


### Editorial Notes.

---

DR. BROWN-SÉQUARD, the great French physician, is dead.

---

THE reunion of the United Confederate Veterans will take place in Birmingham, Ala., on April 25th and 26th.

---

THE Association of Military Surgeons of the United States will meet in Washington, D. C., May 1, 2 and 3.

---

DR. CHAS. N. SMITH, editor of the *American Gynecological Journal*, published at Toledo, Ohio, announces the discontinuance of that magazine.

DR. ROBERT B. CLOUD, of Hephzibah, Ga., favored us with a call a few days ago.

---

IT is with pleasure that we acknowledge the receipt of the first number of the New York *State Medical Reporter*, published in Rochester, N. Y., and edited by Dr. H. Bronson Gee. The *Reporter* is issued monthly and comes laden with many good things.

---

WE desire to call the attention of our readers to the following new advertisements which appear in this issue and hope they will read them: New York Pharmaceutical Company, Bedford Springs, Mass.; Rawley Springs, Rawley, Va.; Dr. George W. Brown's Private Infirmary, Atlanta; Dios Chemical Company (Neurosine) St. Louis, Mo.; Listol Chemical Company, Chicago, Ill.; Labordine Chemical Company, St. Louis, Mo.; Weller-Stephens Saddle Bag Company, St. Louis, Mo.; Paris Medicine Company, St. Louis, Mo.; Chattanooga Medical College.

---

IMPORTANT PATENT DECISION.—The Allen pump patents sustained. Judge Grosscup, of the United States Circuit Court in Chicago, has just rendered a lengthy decision sustaining the validity of the Allen patents. This decision is the result of a suit brought by Mr. Charles Truax, Chicago, against W. C. Carroll, Burton F. Hales *et al.* of the Physicians' National Supply Company, for manufacturing and selling surgical pumps resembling those manufactured under the Allen patents. This is an important decision and one of considerable interest to the medical profession.

---

THE ATLANTA POLYCLINIC.—We have been advised that the faculty of the above institution have decided to close their doors. Though short lived, a great deal of clinical material was at its disposal and much good was accomplished toward relieving the suffering poor of our city. While we were in thorough sympathy and indorsed the aim of this institution, we did not see how it could be a financial success, even within the next few years. Atlanta is a recognized medical center and supports several flourishing medical colleges, but great as she is, a post-graduate school is something to be looked for in the dim and distant future.

